# Humanized Mouse Model as a Novel Approach in the Assessment of Human Allogeneic Responses in Organ Transplantation

**DOI:** 10.3389/fimmu.2021.687715

**Published:** 2021-06-11

**Authors:** Ashwin Ajith, Laura L. Mulloy, Md. Abu Musa, Valia Bravo-Egana, Daniel David Horuzsko, Imran Gani, Anatolij Horuzsko

**Affiliations:** ^1^ Georgia Cancer Center, Department of Medicine, Medical College of Georgia, Augusta University, Augusta, GA, United States; ^2^ Nephrology Division, Department of Medicine, Augusta University, Augusta, GA, United States; ^3^ Histocompatibility and Immunology Laboratory, Department of Surgery, Medical College of Georgia, Augusta University Medical Center, Augusta, GA, United States; ^4^ Program of Osteopathic Medicine, Philadelphia College of Osteopathic Medicine South Georgia, Moultrie, GA, United States

**Keywords:** allogeneic, autologous, donor selection, HLA, humanized mouse, immunogenicity, organ transplantation

## Abstract

The outcome of organ transplantation is largely dictated by selection of a well-matched donor, which results in less chance of graft rejection. An allogeneic immune response is the main immunological barrier for successful organ transplantation. Donor and recipient human leukocyte antigen (HLA) mismatching diminishes outcomes after solid organ transplantation. The current evaluation of HLA incompatibility does not provide information on the immunogenicity of individual HLA mismatches and impact of non-HLA-related alloantigens, especially *in vivo*. Here we demonstrate a new method for analysis of alloimmune responsiveness between donor and recipient *in vivo* by introducing a humanized mouse model. Using molecular, cellular, and genomic analyses, we demonstrated that a recipient’s personalized humanized mouse provided the most sensitive assessment of allogeneic responsiveness to potential donors. In our study, HLA typing provided a better recipient-donor match for one donor among two related donors. In contrast, assessment of an allogeneic response by mixed lymphocyte reaction (MLR) was indistinguishable between these donors. We determined that, in the recipient’s humanized mouse model, the donor selected by HLA typing induced the strongest allogeneic response with markedly increased allograft rejection markers, including activated cytotoxic Granzyme B-expressing CD8^+^ T cells. Moreover, the same donor induced stronger upregulation of genes involved in the allograft rejection pathway as determined by transcriptome analysis of isolated human CD45^+^cells. Thus, the humanized mouse model determined the lowest degree of recipient-donor alloimmune response, allowing for better selection of donor and minimized immunological risk of allograft rejection in organ transplantation. In addition, this approach could be used to evaluate the level of alloresponse in allogeneic cell-based therapies that include cell products derived from pluripotent embryonic stem cells or adult stem cells, both undifferentiated and differentiated, all of which will produce allogeneic immune responses.

## Introduction

Solid organ transplantation has emerged as a lifesaving operation for patients with end-stage organ failure. New advancements in immunosuppressive regimens, HLA typing, and surgery have allowed transplantation of a variety of solid organs, e.g., liver, kidney, lungs, with minimal complication and decreased ischemic injury (1, 2). However, to date solid organ transplantation has not achieved its full clinical potential due to the over reliance on lifelong immunosuppressive regimens to prevent rejection of the graft tissues by the host immune system (3, 4). An efficient transplant depends on balancing the probability of transplant rejection and the side-effects of immunosuppressants that include long-term risk of infection and malignancy (5, 6). One of the main factors to successful organ transplantation is recipient and donor human leukocyte antigen (HLA) compatibility. HLA matching in solid organ transplantation has well-known benefits, including better graft function, longer graft and patient survival, and the possibility to reduce immunosuppressive therapy (7, 8). HLA mismatches are linked to more rejection episodes that require increased immunosuppressive therapies, which affect the function of the transplanted organ and increase the risk of infection and malignancy (7, 9). The identification of HLA mismatches by current methods does not provide information on the immunogenicity of mismatching HLA antigens and, most importantly, their potential to activate immune cells in individual recipient-donor combinations (10). Strategies to optimize organ transplantation, especially for patients with living-related donors, should take into account the assessment of HLA immunogenicity to identify the immunologically best-matched donor when multiple donors available (11, 12). Additionally, HLA typing does not take into account the impact of non-HLA-related alloantigens such as HY, MICA, and PIRCHE II, which have an integral role in initiating allogeneic immune response post-transplant (13–15). We have developed a novel approach to evaluate the level of recipient-donor allogeneic responsiveness *in vivo* using a personalized humanized mouse. Humanized mouse model systems have emerged as an integral research tool for the study of pathological conditions in a stimulated human immune system in the mouse. The humanized mouse system was developed by the systemic progression of genetic modifications on immunodeficient mice. One of the most common immunodeficient strains for research is the NOD (non-obese-diabetic) *scid* (severe-combined immunodeficient) gamma (NOD.Cg-*Prkdc* ^scid^ *Il2rg* ^tm1Wjl^/SzJ) (NSG) mouse (16, 17). An NSG mouse is a non-obese-diabetic mouse model having the *Prkdc* ^scid^ mutation along with a homozygous target mutation at the IL-2R gamma chain locus. These mutations significantly expand the mouse life span while other immunodeficient mice tend to die prematurely due to thymic lymphomas (18). NSG mice demonstrated the highest rate of engraftment, supporting survival and proliferation of human B and T cells (19–21). NSG mice lack innate mature T cells and B cells, have defective NK cell activity, and altered antigenic expression, and thus have the best engraftment rates in comparison to other strains (22–24). This *in vivo* humanized mouse model approach provides the opportunity to determine the lowest degree of recipient-donor alloimmune response, leading to a better selection of the donor and diminishes the HLA-related immunological risk of allograft rejection in organ and bone marrow transplantation. In addition, this approach could be used to evaluate the level of alloimmune responsiveness in allogeneic cell-based therapies that include cell products derived from pluripotent embryonic stem cells or adult stem cells, both undifferentiated and differentiated, all of which will produce allogeneic immune responses.

## Materials and Methods

### Studies Involving Human Subjects

Human subjects were enrolled for the study as per protocol 1598406, approved by the Augusta University Institutional Review Board. Written informed consent was obtained from all subjects participating in the study.

### Inclusion of Identifiable Human Data

No human images or potentially identifiable data are presented in this study.

### HLA Typing

Genomic DNA was isolated from blood samples collected in acid citrate dextrose from volunteer participants in this study. Typing was performed using LinkSēq HLA Typing Real-Time PCR Kit (1580R, One Lambda), following the manufacturer’s protocol. The tray requires a minimum DNA input of 3.7 µg of DNA. In brief, LinkSēq 384-well trays included a variety of sequence-specific primers (SSPs) distributed in each well combined with a fluorescent, double‐stranded DNA‐binding dye (SYBR Green) to identify the presence or absence of amplification products. A real‐time PCR instrument was used to detect these products. Raw fluorescence first derivative (dF) and temperature data are exported from the real‐time PCR instrument for analysis by proprietary SureTyper software (STTPGRX, One Lambda). SureTyper plots dF against temperature to generate a melt‐curve for each reaction well. The melt-curves from each well are examined, and positive and negative peaks are identified. SureTyper compares the pattern of positive and negative reactions against known patterns of amplification for HLA alleles included in the IMGT/HLA database and assigns the most probable alleles. Some infrequent (rare) alleles could not be excluded. The proprietary primers used by LinkSēq cover both intron and exon regions from exons 1 through 7. The array of primers included those necessary to rule out frequent null alleles that are required to be resolved.

### Mice and Generation of Personalized Humanized Mice

All mice were maintained under specific pathogen-free conditions at Augusta University with Institutional Animal Care and Use Committee (IACUC) approval (protocol 2008-0051). Animal studies were performed in strict accordance with recommendations in the NIH Guide for the Care and Use of Laboratory Animals (National Academies Press, 2011). For the development of a humanized mouse model, we used NOD.Cg-Prkdc^scid^ Il2rg^tm1Wjl^/SzJ (NSG) mice from The Jackson Laboratory (005557). NSG mice (5 to 12 weeks old) were given a single intravenous lateral tail injection of different amounts (5.0 x 10^6^, 8.0 x 10^6^, or 10.0 x 10^6^) of human peripheral blood mononuclear cells (PBMCs) from healthy volunteers (recipients). PBMCs were collected in EDTA and purified by Histopaque 1077 (10771, MilliporeSigma) density gradient. To assess the rate of human cell engraftment in the humanized mice, flow cytometry analysis was conducted using blood samples from the humanized mice and control non-engrafted mice. Red blood cells were lysed using ACK lysis buffer (A1049201, Thermo Fisher Scientific) followed by staining of PBMCs with anti-human CD45 (368531, 1:400) and anti-mouse CD45 (109823, 1:400) monoclonal antibodies (mAbs) (all from Biolegend). For studies on human allogeneic responses *in vivo*, a humanized mouse received 5.0 x 10^5^ PBMCs from related or unrelated donors pretreated with Mitomycin C (BP25312, Fisher Scientific) at 50μg/ml, and 25 U/ml recombinant human IL-2 (rhIL-2) (202-IL-010, R&D Systems). On day 5, mice were sacrificed, and human cells were analyzed for the activation of T cells and intracellular expression of Granzyme B, Perforin, IL-2, and IFN-γ. All animals were monitored triweekly for development of graft *versus* host disease (GVHD) and no significant symptoms were observed.

### Antibodies and Flow Cytometry Analysis

For each experimental condition, cells from human PBMCs, mouse peripheral blood, and mouse splenocytes were isolated and labeled with antibodies at 4°C for 45 min in the dark as follows. Anti-human antibodies: CD3 (300412, clone UCHT1, 1:300), CD4 (17-0049-73, clone RPA-T4, 1:300), CD8 (301008, clone RPA-T8, 1:300), CD25 (302606, clone BC96, 1:200), IL-2 (500306, clone MQ1-17H12, 1:300), IFN-γ (502523, clone 4S.B3, 1:300), CD62L (304813, clone DREG-56, 1:300) CD45RA (304110, clone HI100, 1:300); Anti-human/mouse antibody Granzyme B (515403, clone GB11, 1:300); Anti-mouse antibodies: CD3 (100321, clone 145-2C11, 1:300), CD4 (100407, clone GK1.5, 1:200), CD8 (100713, clone 53-6.7, 1:300), and CD25 (10211, clone PC61, 1:200). All antibodies were from Biolegend. All samples were pre-incubated with TruStain fcX (101320, clone 93, 1:100, Biolegend) to block the Fc receptors. Intracellular staining was carried out per the manufacturer’s instruction described in the True Nuclear Transcription Factor Kit (424401, Biolegend). Samples were acquired on the FACS Canto (BD Biosciences) and analyzed using FlowJo version 10.1 (Becton, Dickinson & Company). Dead cells were excluded from the analysis based on the forward and side scatter characteristics.

### Human Transplant Rejection PCR Arrays and Real-Time Quantitative PCR

Spleens from all challenged groups of the humanized mice were harvested, and the hCD8^+^ T cells were isolated using the EasySep Human CD8^+^ T cell isolation kit (17913, Stemcell Technologies). Total RNA was extracted using TRIzol reagent (15-596-026, Thermo Fisher Scientific) followed by purification using RNEasy mini kit (74104, Qiagen). A total of 1 µg high-quality total RNA was then reverse transcribed using the first strand synthesis kit (330404, Qiagen) and subsequently analyzed by the Human Transplant Rejection RT² Profiler PCR Array (PAHS-166Z, Qiagen) in accordance with the manufacturer’s instructions. Qiagen’s online web analysis tool was used to formulate the comparative heat maps, while fold change was determined by calculating the ratio of mRNA levels to control values using the Δ threshold cycle (Ct) method (2^- ΔΔCt^). All data were normalized based on the average of three housekeeping genes, *ACTB*, *GAPDH*, and *HPRT1*. PCR conditions used for the Applied Biosystems Step One plus Real-time PCR system (Applied Biosystems) involved holding for 10 min at 95°C followed by 40 cycles of 15 s at 95°C and 60 s at 60°C. For real-time quantitative PCR (RT-qPCR) analysis, a total of 1 µg of total RNA was isolated and then reverse transcribed using the first strand synthesis kit (330401, Qiagen). 1ng cDNA was then amplified by real-time PCR using primers. Specific primer sequences and expected product size are listed in **Table 1**. Quantification was performed by normalizing the Ct values of each sample to *rRNA*, *ACTB*, *GAPDH*, and *HPRT1*. Values are expressed as fold induction in comparison to the analyzed group. RT-qPCR was performed for 40 cycles of 20 s at 95°C and 30 s at different temperatures for an annealing/extension step using an ABI StepOnePlus™ detection system (Applied Biosystems).

**Table 1:** Primer sequences used for Real-Time qPCR and the expected product size.

Gene	Sequences, 5’-3’		Product size (bp)
	Forward	Reverse	
*GZMB*	CGACAGTACCATTGAGTTGTGCG	TTCGTCCATAGGAGACAATGCCC	122
*PRF1*	ACTCACAGGCAGCCAACTTTGC	CTCTTGAAGTCAGGGTGCAGCG	133
*CD80*	CTCTTGGTGCTGGCTGGTCTTT	GCCAGTAGATGCGAGTTTGTGC	136
*IL2*	AGAACTCAAACCTCTGGAGGAAG	GCTGTCTCATCAGCATATTCACAC	153
*PECAM*	AAGTGGAGTCCAGCCGCATATC	ATGGAGCAGGACAGGTTCAGTC	133
*TAP1*	GCAGTCAACTCCTGGACCACTA	CAAGGTTCCCACTGCTTACAGC	109
*CTLA4*	ACGGGACTCTACATCTGCAAGG	GGAGGAAGTCAGAATCTGGGCA	121
*CD86*	CCATCAGCTTGTCTGTTTCATTCC	GCTGTAATCCAAGGAATGTGGTC	154
*GAPDH*	GTCTCCTCTGACTTCAACAGCG	ACCACCCTGTTGCTGTAGCCAA	131
*ACTB*	CACCATTGGCAATGAGCGGTTC	AGGTCTTTGCGGATGTCCACGT	135
*TNF*	CTCTTCTGCCTGCTGCACTTTG	ATGGGCTACAGGCTTGTCACTC	135
*TIMP1*	GGAGAGTGTCTGCGGATACTTC	GCAGGTAGTGATGTGCAAGAGTC	111
*HPRT1*	CATTATGCTGAGGATTTGGAAAGG	CTTGAGCACACAGAGGGCTACA	129

### Human Transcriptome and Data Analysis

Human cells were isolated from splenocytes of humanized mice by density centrifugation, followed by MACS magnetic bead separation of human CD45^+^ cells (130-045-801, Miltenyi Biotec). Total RNA was isolated as described above. RNA purity and concentration were evaluated by spectrophotometry using NanoDrop ND-1000 (Thermo Fisher Scientific). RNA quality was assessed by the Agilent 2200 TapeStation (Agilent Technologies) and assured of an RNA Integrity Number (RIN) ≥ 7. The Human Gene 2.0 ST array 4 (Applied Biosystems), which covers 24,838 genes, was used for gene expression profiling. Total RNA samples were processed using the GeneChip WT PLUS Reagent Kit (Applied Biosystems). Briefly, the WT PLUS Reagent Kit was used to generate sense strand cDNAs using 250 ng of starting RNA material. The synthesized sense strand cDNAs (5.5 µg) were fragmented, biotin-labeled, and hybridized onto the arrays according to the manufacturer’s protocol. After 16 hours of hybridization, the arrays were washed and stained using the Affymetrix GeneChip Fluidics Station 450 system. The stained arrays were scanned on an Affymetrix GeneChip Scanner 3000. Data were obtained in the form of CEL files, which were imported into Partek Genomic Suites version 6.6 (Partek) using the standard import tool with a Robust Multi-array Average (RMA) normalization. Differential expression was calculated using ANOVA of Partek Package and filtered with a *p*-value cutoff of 0.05 and fold-change cutoff to screen out the differentially expressed genes in each comparison. The significant gene list was used to generate a hierarchical clustering plot by the standardized expression values. Further analysis for the Venn diagram and signaling pathway was carried out using the Transcriptome Analysis Console 4.0 (TAC 4.0) (Applied Biosystems). Additional RT-qPCR was performed as described above.

### Immunohistochemistry

The groups of humanized mice were sacrificed 5 days post-challenge and their spleens harvested. Immunohistochemistry was performed as previously described (25). Briefly, spleen tissues were fixed in 4% paraformaldehyde and embedded with paraffin. 7μm sections were boiled in citrate buffer antigen retrieval solution containing 10mM sodium citrate and 0.05% Tween 20 (Thermo Fisher Scientific) for 30 minutes and washed twice with PBS. Spleen sections were blocked with 3% bovine serum albumin (Thermo Fisher Scientific) in PBST for 1 hour before incubation with primary antibodies. Incubation was carried out in the presence of FITC-conjugated anti-human CD8 (344703, Biolegend) and PE-conjugated anti-human Granzyme B (396405, Biolegend) for 1 hour at room temperature in a dark humid chamber. Sections were washed twice with PBS, and nuclei were visualized by mounting in medium containing DAPI (H-1500, Vector Laboratories). All images were taken on a Keyence BZ-X800 microscope and analyzed with the BZ-X800 image viewer (Keyence).

### Mixed Lymphocyte Reaction (MLR)

The recipient PBMCs acting as responders were labeled with CFSE (65-0850-84, eBioscience) at 2μM. PBMCs prepared from related and unrelated donors and autologous cells serving as stimulator cells were treated with Mitomycin C (BP25312, Fisher Scientific) at 50μg/ml for 35 minutes. Both responder and stimulator cells at a ratio of 1:1 were co-cultured in complete RPMI (Gibco) at 37°C in the dark for 3 days. Thereafter, proliferation of the CFSE-labeled responder cells was analyzed by flow cytometry, and cells were stained with anti-CD8, -CD4, -CD25, -Granzyme B, -IL-2, and -IFN-γ antibodies (Biolegend). Pro-inflammatory cytokine profiles of the CD8^+^CD25^+^GranzymeB^+^ cytotoxic cells were analyzed using FlowJo version 10.1 (Becton, Dickinson & Company). All experiments were run in triplicate and acquired on the Attune NxT Flow Cytometer (Thermo Fisher Scientific).

### Statistical Analysis

All data are expressed as mean ± SD. Comparisons of 2 groups were analyzed using an unpaired, 2-tailed Student’s *t*-test using GraphPad Prism (GraphPad Software). One-way ANOVA was used for comparing multiple groups. For pathway analysis of transcriptome array significance was calculated using 2-sided Fisher’s exact test. A *p*-value of less than 0.05 was considered statistically significant.

## Results

### Assessment of Immunocompatibility Between Recipient and Donors by Conventional HLA Typing

Conventional HLA typing was carried out to assess the immunocompatibility between a recipient (R) and two related donors (RD1 and RD2) and an unrelated donor (UD). In this case, the R was male, with no history of blood transfusions. RD1 and UD were males and RD2 was female. The HLA typing was performed by the Tissue Typing Services of Medical College of Georgia (MCG) following the protocols mandated for typical solid organ transplantation. As shown in **Table 2**, R and RD1 shared a haplotype in one HLA-A, one HLA-B, one HLA-C, two HLA-DRB1, two HLA-DQA1, two HLA-DQB1, one HLA-DPA1, and one HLA-DPB1 antigen match as well as one HLA-DRB4, one HLA-DPA1, and one HLA-DPB1 antigen mismatch. We determined that R shares the other haplotype with RD2, which are one HLA-A, one HLA-B, one HLA-C, two HLA-DRB1, one HLA-DRB4, one HLA-DRB5, two HLA-DQA1, two HLA-DQB1, two HLA-DPA1, and two HLA-DPB1 antigen matches. In contrast to RD1 and RD2, UD has less immunocompatibility with R, showing mismatch with almost all typed HLA antigens, except matches with one HLA-DRB5, one HLA-DQA1, one HLA-DPA1, and one HLA-DPB1 antigens. Based on the HLA typing results, the most compatible and preferential donor for organ transplantation for recipient R would be RD2, with RD1 being a close match and UD being incompatible.

**Table 2:** HLA-typing results of recipient, related donors, and unrelated donor.

Sample ID	Relation	HLA-A*	HLA-B*	HLA-C*	HLA-DRB1*	HLA-DRB3*	HLA-DRB4*	HLA-DRB5*	HLA-DQA1*	HLA-DQB1*	HLA-DPA1*	HLA-DPB1*	Haplotype Assignment	Match
R	Recipient	03:01	35:01	04:01	07:01	Not present	01:03	Not present	01:02	02:02	01:03	04:01	a	N/A
		25:01	18:01	12:03	15:01	Not present	Not present	01	02:01	06:02	01:03	02:01	c	
RD1	Related	29:02	14:02	08:02	07:01	Not present	01:01	Not present	01:02	02:02	01:04	15:01		1A, 1B, 1C, 2DR, 2DQ,1DP
		25:01	18:01	12:03	15:01	Not present	Not present	01	02:01	06:02	01:03	04:01	c	
RD2	Related	11:01	56:01	01:02	15:01	Not present	Not present	01	02:01	06:02	01:03	02:01		1A, 1B, 1C, 2DR, 2DQ,2DP
		03:01	35:01	04:01	07:01	Not present	01:03	Not present	01:02	02:02	01:03	04:01	a	
UD	Unrelated	11:01	52:01	12:02	14:04	02:02	Not present	Not present	01	05:03	01:03	04:01	Not applicable	1DR, 1DQ
		26:01	40:06	15:02	15:01	Not present	Not present	01	01:02	06:01	02:01	14:01		

### Assessment of the Recipient Alloimmune Response to Related and Unrelated Donor Alloantigens by MLR

**Figure 1:**
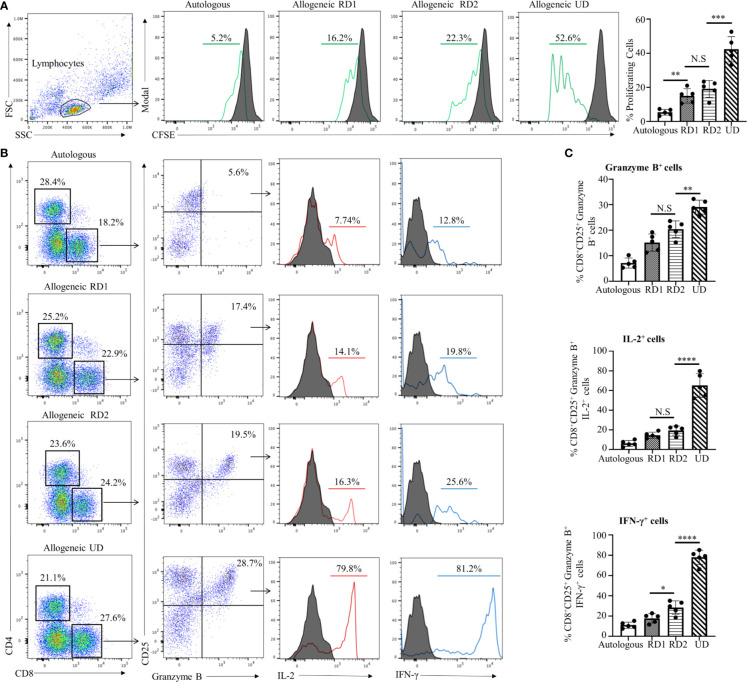
Recipient’s allogeneic response generated in MLR was indistinguishable between RD1 and RD2 donors. **(A)** Representative flow cytometry histogram depicts CFSE-based proliferation of recipient cells in response to alloantigens from related (RD1, RD2) and unrelated (UD) donor stimulator cells. Filled histograms show CFSE-stained non-proliferating control, and green histograms show CFSE-stained proliferating cells. Graphical summary illustrates frequency (%) of proliferating recipient cells with each stimulator group. Data are representative of 5 separate experiments. Data presented as mean ± SD. **p < 0.01, ***p < 0.001, NS., not significant. **(B)** Representative flow cytometry color plots depict gating strategy for the identification of CD25^+^ Granzyme B-expressing CD8^+^ T cells amongst the responding recipient cells in each stimulator group. Histogram illustrates expression of pro-inflammatory cytokines IL-2 (red line) and IFN-γ (blue line) in the cytotoxic CD8^+^CD25^+^Granzyme B^+^ cells. Filled histogram shows isotype control. **(C)** Graphical summary depicts frequency (%) of CD8^+^CD25^+^Granzyme B^+^ cells. The IL-2 and IFN-γ profile of CD8^+^CD25^+^Granzyme B^+^ in each stimulator group is depicted. Data presented as mean ± SD from 5 experiments. *p < 0.05, **p < 0.01, ****p < 0.0001, NS., not significant.

MLR is one of the *in vitro* methods to determine the allogeneic responsiveness that is caused by the recognition of HLA-related and non-HLA-related alloantigens provoking a T-cell-mediated immune response. Proliferative responses of recipient cells to alloantigens were measured by flow cytometry with CFSE-labeled responder cells. The stimulator cells from all donors and in autologous combinations were Mitomycin C-treated cells to provide one-way recognition of alloresponse only. As shown in **Figure 1A**, proliferation of CFSE-labeled recipient responder cells was least in an autologous combination (5.3 ± 2.0%, *p*<0.01). However, the recipient responder cells demonstrated a robust proliferation (42.3 ± 7.4%, *p*<0.001) to the allogeneic UD cells. In contrast, the proliferation of recipient cells to the RD1 (15.0 ± 4.2%) and RD2 (19.0 ± 5.1%) allogeneic cells was moderate and there was no significant difference (*p*=0.20) between the groups (**Figure 1A**). The total number of CD8^+^ T cells was increased in both RD1 and RD2 allogeneic responses with a marked increase in the allogeneic UD response (**Figure 1B**, left panels). In addition, an elevated number of activated, Granzyme B-expressing CD8^+^ T cells was determined in allogeneic RD1 (17.1 ± 3.6%), RD2 (20.3 ± 3.2%), and UD (29.0 ± 2.7%) responses. However, no statistical difference (*p*=0.07) was determined between RD1 and RD2 allogeneic immune responses (**Figures 1B, C**). The analysis of IL-2 (77.0 ± 7.0%, *p*<0.001) and IFN-γ (65.0 ± 12.0%, *p*<0.001) production in activated Granzyme B-positive CD8^+^ T cells revealed robust expression of these cytokines in the allogeneic UD response, with a moderate increase in RD1 and RD2 responses, and only IFN- γ was augmented in the RD2 (25.0 ± 6.0%, *p*<0.05) compared to the RD1 (17.7 ± 4.6%) response (**Figures 1B, C**). These data suggested that allogeneic responses in MLRs demonstrated a recipient’s strong alloresponse to the UD donor cells. However, the recipient’s allogeneic response to cells from two related donors was moderate and was insufficient to determine differences in immunogenicity between the two donors.

### Generation and Optimization of Humanized Mouse for Assessment of Allogeneic Response *In Vivo*

**Figure 2:**
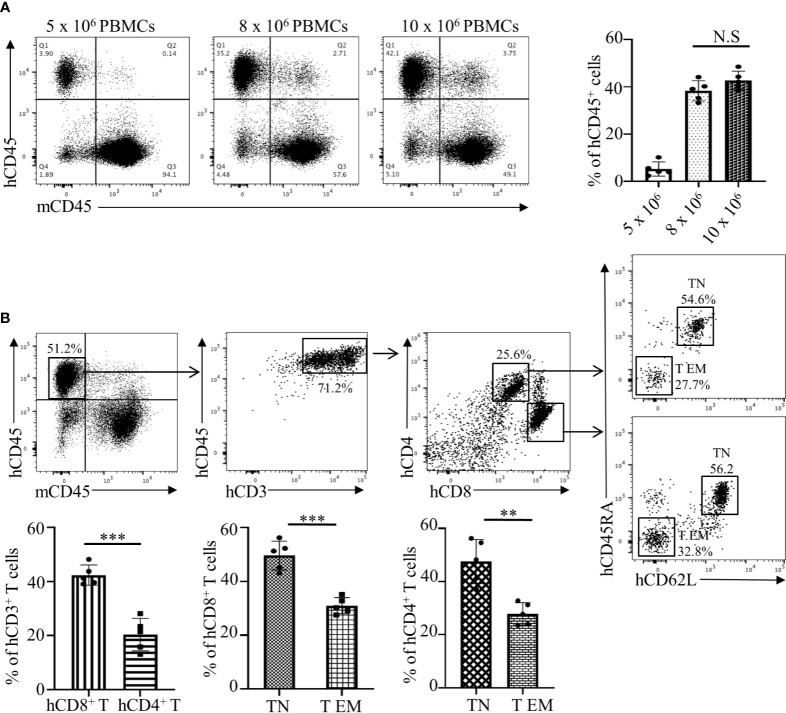
Analyses and optimization of humanization phase in the Hu-NSG-PBMC mouse model. **(A)** Representative flow cytometry dot plots depict engraftment of hCD45^+^ cells in the humanized mouse 3 weeks after initial dose of 5, 8, or 10 x 10^6^ recipient PBMCs. Graphical summary illustrates frequency (%) of recipient hCD45^+^ cells in the spleen of the humanized mouse (n=5 mice per group). Data presented as mean ± SD. NS., not significant. **(B)** Flow cytometry dot plots depict phenotype of the engrafted hCD45^+^ cells in the humanized mouse with gating for hCD3^+^, hCD4^+^, and hCD8^+^ T cell populations. Characterization of hCD4 and hCD8^+^ T cells was expanded showing their respective CD62L and CD45RA expression. Graphical summary depicts frequency (%) of hCD4^+^ and hCD8^+^ T cells as well as percent of TN (CD62L^+^CD45RA^+^) and T EM (CD62L^-^CD45RA^-^) populations. n=5 mice per group. Data presented as mean ± SD. **p < 0.01, ***p < 0.001.

The assessment of an allogeneic response in MLR is limited due to several factors that include an artificial *in vitro* environment and manipulations with stimulator cells. Therefore, we have developed an *in vivo* humanized mouse model that is similar to the allogeneic response induced during human organ transplantation or allogeneic cell-based therapies. We used the NOD-*scid* IL2Rgamma null (NSG) mouse that has been represented as the gold standard for studies involving human hematopoietic cells. First, we investigated the NSG mouse engraftment potential for human PBMCs, which is an essential factor for the humanization phase. We optimized the number of cells required to obtain efficient engraftment of human PBMCs. Flow cytometry analysis of human CD45^+^ cells from the spleen of humanized mice demonstrated that injections of 8 x 10^6^ and 10 x 10^6^ cells have the strongest engraftment of human PBMCs compared to 5 x 10^6^ cells. Moreover, no statistical differences were determined between groups of mice injected with 8 x 10^6^ or 10 x 10^6^ cells (**Figure 2A**, *p*>0.5). Therefore, the optimal number of 8 x 10^6^ PBMCs from the recipient was used in further studies. We observed that, post humanization at the 3^rd^ week, almost 71.2% of engrafted hCD45^+^ cells were CD3^+^ T cells with a CD4:CD8 ratio of 1:2 (**Figure 2B**). This predisposition towards amplified CD8^+^ T cell expansion has been previously reported and is commonly observed in the NOD background due to greater selective interaction between murine MHC Class I molecules and human CD8^+^ T cells (26–28). However, despite their selective proliferation, a significant majority of the engrafted CD8^+^ T cells (49.2 ± 6.3%) showed a naïve T cell (TN) phenotype (CD62L^+^CD45RA^+^) while only 30.0 ± 3.1% showed an effector memory (T EM) phenotype with a CD62L^-^CD45RA^-^ expression profile (**Figure 2B**). A similar pattern was observed amongst CD4^+^ T cells as well, where the majority of 45.1 ± 8.0% cells maintained a naïve phenotype (**Figure 2B**). However, on the 4^th^ week there is a dynamic transition in the TN and T EM populations with decreasing naïve cells making the 3 weeks of PBMC reconstitution optimal for the study (**Supplementary Figure 1**). These results showed that the NSG mouse allowed efficient engraftment of human PBMCs while maintaining lymphocytes in a naïve state prime for activation in response to suitable stimuli. These observations make our humanized-NSG-PBMC model an efficient novel method for investigating allogeneic immune responses between recipient and donors.

### Humanized Mouse Model Provides a More Sensitive Assessment of Allogeneic Response

#### Hu-NSG-PBMC Mice Exhibit Robust CD8^+^ T Cell-Driven Allogeneic Response

**Figure 3:**
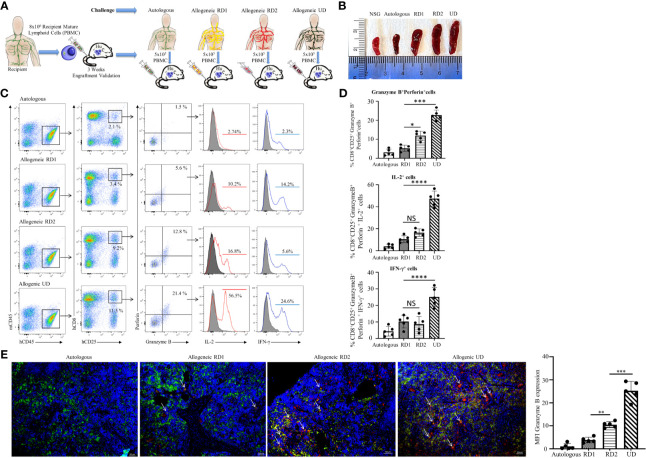
Hu-NSG-PBMC mouse model exhibits strong CD8^+^ T cell-mediated allogeneic immune response. **(A)** Experimental design schematic illustrates humanization of the NSG mouse with PBMCs from the recipient followed by challenge phase with autologous control, allogeneic related donors (RD1, RD2), or allogeneic unrelated donor (UD) PBMCs. **(B)** Representative spleens isolated from the different challenge groups are shown. **(C)** Representative flow cytometry color plots depict gating strategy for CD25^+^ Granzyme B and Perforin expressing activated cytotoxic hCD8^+^ T cells in spleen of the humanized mouse for the challenge groups. Histogram shows expression of pro-inflammatory cytokines IL-2 (red line) and IFN-γ (blue line) amongst these cytotoxic hCD8^+^T cells. **(D)** Graphical summary depicts the frequency (%) of hCD8^+^CD25^+^Granzyme B^+^Perforin^+^ cells. The IL-2 and IFN-γ profile of CD8^+^CD25^+^Granzyme B^+^ Perforin^+^ cells in each challenge group is depicted (n=5 mice per group). Data presented as mean ± SD. *p < 0.05, ***p < 0.001, ****p < 0.0001. NS., not significant. **(E)** Representative immunofluorescent microscopy of spleen sections of the humanized mouse stained with FITC-conjugated hCD8, PE-conjugated hGranzyme B and nuclear staining shown by DAPI (scale bar 5μm). Graphical summary shows mean fluorescence intensity (MFI) for hGranzyme B expression amongst the challenge groups. n=5 mice per group. Data presented as mean ± SD of at least 3 sections from each mouse. **p < 0.01, ***p < 0.001.

Post humanization and engraftment validation, the Hu-NSG-PBMC mice were subjected to an allogeneic challenge either by PBMCs from an unrelated donor (UD) or donors (RD1 and RD2) that were closely related to the recipient. This challenge was induced by tail vein injection of 5 x 10^5^ PBMCs to test the efficiency of the newly engrafted human immune cells to induce an appropriate allogeneic response. An autologous challenge with the volunteer’s own PBMCs served as the control (**Figure 3A**). In the allogeneic challenge, the UD PBMCs induced significant splenomegaly with massive cell infiltration at 5 days of the challenge. The unchallenged control NSG mice and the autologous challenged mice showed minimal or no splenomegaly while the RD1 and RD2 mice presented enlarged spleens (**Figure 3B**). Additional flow cytometry analysis of the spleen showed a significant activation of the infiltrated hCD8^+^ T cells, with 15% showing CD25 expression in the UD group while RD1 induced 3% and RD2 induced 9.2% activation. However, 21.5 ± 2.8% of the activated hCD8^+^CD25^+^ T cells in the UD group showed a cytotoxic phenotype with positive expression for Perforin and Granzyme B as well as expression of pro-inflammatory cytokines IL-2 and IFN-γ (**Figures 3C, D**). Amongst the related donors RD2 challenge elicited a significantly higher cytotoxic phenotype (13.2 ± 2.4%, *p*<0.01) from the infiltrated hCD8^+^ T cells in comparison to RD1 (6.2 ± 2.1%) (**Figure 3D**). The autologously challenged mice induced negligible hCD8^+^ T cell activation and a very minimal cytotoxic phenotype (4 ± 1.7%). These observations were confirmed with immunofluorescence microscopy of spleen sections of the corresponding challenge groups. As shown, we observed maximal infiltration of Granzyme B-expressing hCD8^+^ T cells within the UD (25.2 ± 4.0%, *p*<0.001), minimal infiltration with RD2, and negligible infiltration with RD1 and the autologous control (**Figure 3E**). The Hu-NSG-PBMC mouse model showed a selective cytotoxic hCD8^+^ T cell-mediated allogeneic response to the RD2 stimuli, while at the same time it exhibited a tempered and controlled reaction to the RD1 challenge. These observations establish that our Hu-NSG-PBMC humanized mouse model is immunologically sensitive and specific to resolve the allogeneic stimulus exhibited between related donors and is more reflective of the actual immunocompatibilty with the recipient.

#### Differential Expression of Transplant Rejection Genes in CD8^+^ T Cells Between Two Related Donors

**Figure 4:**
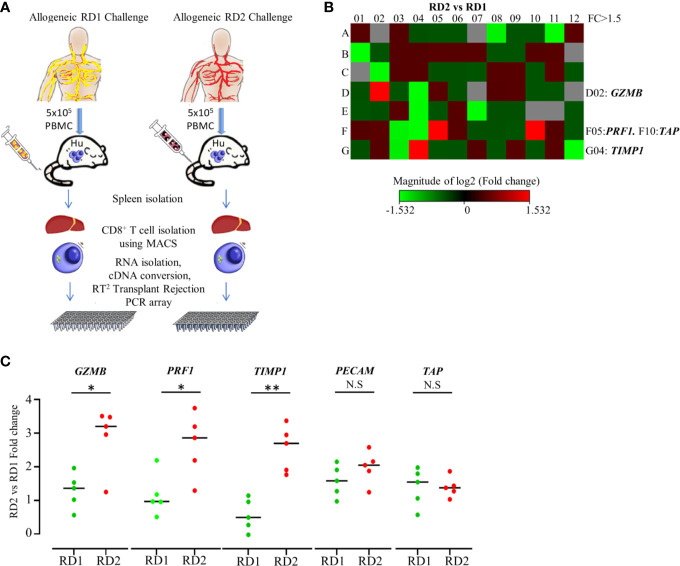
Transplantation rejection genes are significantly upregulated in recipient’s CD8^+^ T cell’s allogeneic response to RD2. **(A)** Experimental design schematic illustrates allogeneic challenge of the humanized mouse with cells from RD1 and RD2 followed by spleen extraction, isolation of infiltrating hCD8^+^ T cells, and transcript analysis using RT^2^ Transplant Rejection PCR array. The gene expression was normalized to the average of three housekeeping genes (*ACTB*, *GAPDH*, *HPRT*), and expression of each gene relative to RD1 is depicted. **(B)** Heat map illustrates fold change of transplant rejection-specific genes in hCD8^+^ T cells of the allogeneic RD2 group in comparison to the RD1 challenge. Red indicates increased and green indicates decreased expression. **(C)** The specific gene expression patterns were confirmed by custom real-time PCR. Gene expression was normalized to *GAPDH* levels, and fold change in mRNA levels of each gene in the RD2 group compared to RD1 is shown. n= 5 mice per group. Data presented as mean ± SD.*p < 0.05, **p < 0.01. NS., not significant.

We established that the engrafted human immune cells of the Hu-NSG-PBMC mice were able to induce a robust immune response to the various allogeneic challenges. In order to delineate the differences in allogeneic responses mediated specifically by hCD8^+^ T cells between the RD1 and RD2 challenges, we performed transcriptional analysis using the RT^2^ Human Transplantation Rejection array. We compared the expression profile of 84 key genes involved in transplant rejection specifically for hCD8^+^ T cells between the RD2 vs RD1 allogeneic challenges (**Figure 4A**). The main aim of this study was to pinpoint selective markers being up-regulated during related donor graft rejections. Our heat map data showed a greater than > 1.5-fold up-regulation of specific genes including *GZMB*, *PRF1*, and *TIMP1* within the RD2 challenge mice in comparison to RD1 (**Figure 4B**). The *GZMB* and *PRF1* transcript increases were statistically confirmed in the RD2 group by additional real-time qPCR analyses using custom primers (**Figure 4C**). These transcriptional results corroborate our earlier observations in which activated hCD8^+^ T cells in the UD allogeneic challenge show similar expression patterns. These observations suggest that the Hu-NSG-PBMC model is capable of mounting an effective immune-activated, cytotoxic response to allogeneic stimuli derived from related donors.

#### Comparison of Transcription Profile of Allogeneic Immune Response Generated in MLR and Hu-NSG-PBMC Mouse Model

**Figure 5:**
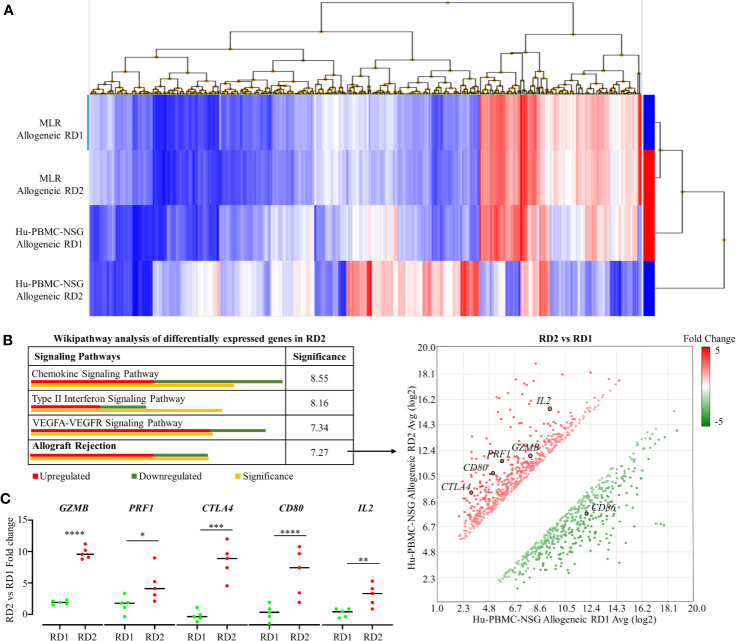
Transcriptome analyses of recipient’s allogeneic responses generated in humanized mice demonstrate differences between related donors, which are indistinguishable in the MLR. **(A)** Human Gene 2.0 ST array was used to plot a heat map representing differential gene expression in hCD3^+^ T cells of the allogeneic RD1 and RD2 challenges for the MLR and the humanized mouse models. Hierarchical clustering was used to segregate individual gene expression in each group into reduced expression (blue) and over-expression (red) conditions. **(B)** Transcriptome Analysis Console was used to carry out pathway analysis on the differentially expressing genes in RD2 challenges of the humanized mouse model. The allograft rejection pathway had 12 significantly upregulated and 7 significantly downregulated genes depicted in the scatter plot. Significance was calculated using 2-sided Fisher’s exact test. **(C)** Custom RT-qPCR was carried out to confirm fold change of target genes integral for allograft rejection comparing the RD2 *vs* RD1 allogeneic challenges. n = 5 mice per group. Data presented as mean ± SD. *p < 0.05, **p < 0.01, ***p < 0.001, ****p < 0.0001.

To demonstrate the efficacy of our novel humanized mouse model for identifying the best potential donor for the recipient, we carried out a transcriptome analysis comparing the allogeneic immune response in MLR and the Hu-NSG-PBMC mouse model (29). Using the GeneChip Human Gene 2.0 ST array, we compared the expression of up to 24,838 genes between the responder hCD3^+^ T cells in the MLR and the infiltrating hCD3^+^ T cells of the Hu-NSG-PBMC mouse model. In the MLR, CD3^+^ T responder cells to RD1 and RD2 challenge showed an identical transcriptional expression profile, clearly demonstrating the inefficiency of MLR in distinguishing the immunogenicity of closely related donors (RD1, RD2) (**Figure 5A**). In contrast, the Hu-NSG-PBMC model showed a significant difference in the transcription expression profile of allogeneic RD2 challenge compared to RD1. We further enriched this set of differentially expressed genes using pathway analysis and observed that allograft rejection was one of the significantly upregulated pathways in the RD2 immune response (**Figure 5A**). Moreover, several clinically relevant markers of graft rejection, such as *GZMB*, *PRF1*, and *IL2*, were significantly up-regulated in RD2 in comparison to RD1 (30, 31). In addition, increased *CTLA4* and *CD80* expression amongst the RD2-induced immune cells point towards an exhausted T cell subpopulation arising after the initial allogeneic immune response (**Figure 5C**) as reported previously (32). Overall, the humanized mouse model of allogeneic response represents a more clinical relevance for donor selection than MLR.

## Discussion

Immunological graft rejection originates from alloimmune T cells derived from the host immune system. The host alloimmune T cell response against donor-derived antigens such as HLA are much stronger in comparison to classical immune responses against pathogen or self (33, 34). This is due to the highly polymorphic nature of HLAs combined with the presence of multiple HLA loci (HLA-A, B-class I antigens; HLA-DR, DQ, DP- class II antigens) (35–37). A diverse mismatch across the HLA alloantigens between the recipient and donor can elicit a robust allogeneic immune response (38, 39). These allospecific T cells of the host immune system can form activated cytotoxic Granzyme B-expressing CD8^+^ T cells that are primarily responsible for graft tissue destruction and transplant failure (40, 41). Therefore, selection of the most immunocompatible donor for the recipient with the least amount of HLA mismatches can maximize graft survival and reduce dependency on immunosuppressive treatments (42, 43). In this scenario, a well-matched related donor is the perfect candidate, allowing adequate time for investigating the level of histocompatibility with the recipient. HLA typing and MLR are the long-time clinical standards for the assessment of donor-recipient immunocompatibility in organ transplantation (44–47). Various studies have established that an increased number of matched antigens and decreased number of mismatched antigens lead to improved graft survival (48–50). However, HLA typing results are often confounded by the varying immunogenicity of the different HLA loci. HLA-DR mismatches are known to contribute heavily to graft rejection, and HLA-A and B matches are also crucial for graft acceptance (7). Meanwhile, HLA-DQ mismatch has no clinical significance unless it is compounded by the presence of a DR mismatch; DP mismatches only become relevant during regraft scenarios (51). Current donor organ allocation strategies consider mismatches at HLA-A, B, and DR to be equally important. However, mounting evidence suggests that each HLA mismatch contributes differently to graft survival; some HLA mismatches look more permissible than others (9, 38, 52). Benefits of dependence on HLA matching are further diluted by other factors such as age; a younger donor age can compensate for the impact of HLA mismatches (53, 54). However, HLA typing does not consider the impact of non-HLA-related alloantigens (55). Growing evidence suggests that as much as 38% of kidney allograft rejections are due to these non-HLA-related alloantigens in comparison to 18% caused by HLA mismatches (14, 56). Thus, there is a need for additional methods to assess immunocompatibility between recipient and donor. The most common and routinely used solution is MLR (57). CFSE-based MLR assays enabled the phenotypic characterization of alloimmune T cells developed by the recipient on stimulation by donor immune cells. However, the correlation between clinical outcomes and *in vitro* functional MLR assays has been low due to the inherent inability of *in vitro* assays to replicate the *in vivo* physiological environment. For example, unresponsive donor reactive cells commonly seen in MLR may arise due to deletion or anergy, a phenomenon that is indistinguishable in an *in vitro* setting (45). Although classical MLR has helped in histocompatibility assessment and pre-transplant risk evaluation, its fundamental disadvantage as an *in vitro* model prevents its translation into distinguishable clinical outcomes (58, 59). Thus, to overcome the limitations of HLA typing and MLR, we have developed a novel *in vivo* humanized mouse model. This model takes into account the varying immunogenicity of HLA antigens as well as the impact of non-HLA-related alloantigens that are ignored during HLA typing. In addition, being an *in vivo* model, we avoided artificial culture conditions and growth factors essential for *in vitro* assays, allowing for a more dynamic and natural background for an allogeneic immune response. The NSG mouse used in this model has defective VDJ recombination and a mutation in the IL-2R gamma chain, allowing for maximal engraftment of human immune cells and making it the most amenable strain for studying allogeneic immune responses (20, 60–63). Advantageously, there is a natural predisposition for the NOD background strains to encourage higher hCD8^+^ T cell engraftment, thus making NSG mice perfect for studying graft rejection (64). In this study, we concentrated mainly on CD8^+^ T cells that are primarily responsible for Granzyme B release and subsequent graft destruction. The future analysis of other immune cells involved in allogeneic response, such as antigen presenting cells (APCs) and CD4^+^ T cells, could be envisaged. For humanization, we utilized the recipient’s PBMCs since they are mature cells capable of providing a fast and robust model for allogeneic immune responses (23, 24, 65). The disadvantage of this model is the GVHD symptoms that can arise during humanization and engraftment of the recipient PBMCs, with maximum development at 6-8 weeks (16, 22, 66, 67). Though, our entire experiment including the humanization and the challenge phase was completed within 26 days. This swift approach in our study allows us a small window of opportunity to assay allogeneic responses while avoiding the significant symptoms of GVHD. As such, this protocol was established after rigorous preliminary studies titrating both the required PBMCs for humanization and the number of donor cells needed for the challenge. Furthermore, in the challenge phase of the experiment, all groups (including autologous, related donor 1, related donor 2, and unrelated donor) were subjected to similar levels of initial GVHD due to the recipient PBMC humanization phase, and hence we have minimized the influence of such background noise in our overall interpretation of the data. As our model makes use of recipient PBMCs prior to transplantation they would not be under any immunosuppressive drug regimens that might affect the reconstitution rates during the humanization phase. As such reconstitution rates for recipients in NSG mice are consistent and, by utilizing 5 mice per group in the challenge phase of our study, we have minimized some of the variations caused by mouse health factors. In the Hu-NSG-PBMC model, the main pathway for allogeneic immune response would be direct allorecognition wherein intact alloantigens presented by donor APCs activate recipient T cells. However, we do not exclude the indirect recognition pathway because recipient APCs are part of the PBMC-NSG mouse system during reconstitution phases and may contribute to the immune response during the challenge phase.

In our histocompatibility study, we showed the most compatible and preferential donor to be RD2, with RD1 being a close match. Predictably, unrelated donor (UD) was the most incompatible. The determining factor was that RD1 had only 8 HLA matches with the recipient while RD2 had 9 HLA matches. As both donors were of relatively similar young ages, in a standard clinical scenario, the preferential donor for organ transplantation could be RD2. The MLR studies showed indistinguishable responses from the recipient immune cells between RD1 and RD2. CFSE proliferation rates were minimal, and cytotoxic CD8^+^ T cell activation and pro-inflammatory cytokine profiles, including IL-2 and IFN-γ, were similar between RD1 and RD2 allogeneic responses. However, the validity of MLR was confirmed by observations with the UD challenge. To overcome the limitations of MLR and HLA typing, we generated the personalized humanized mouse model. Three weeks after the PBMC injection, we determined efficient engraftment of hCD45^+^ cells in the humanized mouse. The NSG mouse showed minimal signs of GVHD with the majority of cells retaining a naïve phenotype with a CD62L^+^CD45RA^+^ expression profile (68). These observations confirmed the presence of a sizeable majority of naïve CD8^+^ T cells unaffected by GVHD that are capable of mounting an allogeneic immune response. Our allogeneic challenges, specifically RD2 and UD, elicited a robust immune response from the humanized mouse. There was increased splenomegaly with RD2 by infiltration of cytotoxic CD8^+^ T cells expressing Perforin and Granzyme B. These alloimmune T cells showed a pro-inflammatory IL-2 and IFN-γ expression profile as well. In comparison, RD1 had minimal splenomegaly and only 2 – 5% infiltration of cytotoxic CD8^+^ T cells. However, this response was still higher than the autologous challenge. In concordance, our Transplant Rejection PCR array analysis on these infiltrating hCD8^+^ T cells identified *PRF1* and *GZMB* as potential identifiers for distinguishing related donor immune responses. The two markers were significantly higher in RD2, in comparison to RD1, both transcriptionally and on flow cytometry analysis. Additionally, our transcriptome array analysis demonstrated a significantly higher allograft rejection-based transcription expression profile of human immune cells in the RD2 challenge by using the Hu-NSG-PBMC model, which was not distinguished in MLR. Thus, our model provides a more sensitive assessment of allogeneic immunogenicity, making RD1 the preferred donor with the additional possibility of reducing the immunosuppressive dosage. Using molecular, cellular, and genomic analyses, we demonstrated that the recipient’s personalized humanized mice provided the most sensitive assessment of allogeneic responsiveness to the potential donors. In addition, this approach could be used to evaluate the level of alloimmune responsiveness in bone marrow transplantation and allogeneic cell-based therapies that include cell products derived from pluripotent embryonic stem cells or adult stem cells, both undifferentiated and differentiated, all of which will produce allogeneic immune responses.

## Data Availability Statement

The datasets presented in this study can be found in online repositories. The names of the repository/repositories and accession number(s) can be found below: ArrayExpress database at EMBL-EBI (www.ebi.ac.uk/arrayexpress) under accession number E-MTAB-10343.

## Ethics Statement

Human subjects were enrolled for the study as per protocol 1598406, approved by the Augusta University Institutional Review Board. Written informed consent was obtained from all subjects participating in the study. The patients/participants provided their written informed consent to participate in this study. All mice were maintained under specific pathogen-free conditions at Augusta University with IACUC approval (protocol 2008-0051). Animal studies were performed in strict accordance with recommendations in the NIH Guide for the Care and Use of Laboratory Animals (National Academies Press, 2011).

## Author Contributions

AH and LM designed the study. AA, DH, VB-E, and MM performed the experiments and analysed the data. IG and VB-E contributed to the interpretation of the data. AH and AA wrote the manuscript. All authors contributed to the article and approved the submitted version.

## Funding

This study was supported by research funding from the Carlos and Marguerite Mason Trust, and by U.S. National Institutes of Health, National Cancer Institute Grant CA 172230 (to AH).

## Conflict of Interest

The authors declare that the research was conducted in the absence of any commercial or financial relationships that could be construed as a potential conflict of interest.
