# Supplementary figures and images for: Humanized Mouse Model as a Novel Approach in the Assessment of Human Allogeneic Responses in Organ Transplantation
Source: Front Immunol. 2021 Jun 11;12:687715. doi: 10.3389/fimmu.2021.687715 (PMC8226140; doi:10.3389/fimmu.2021.687715)

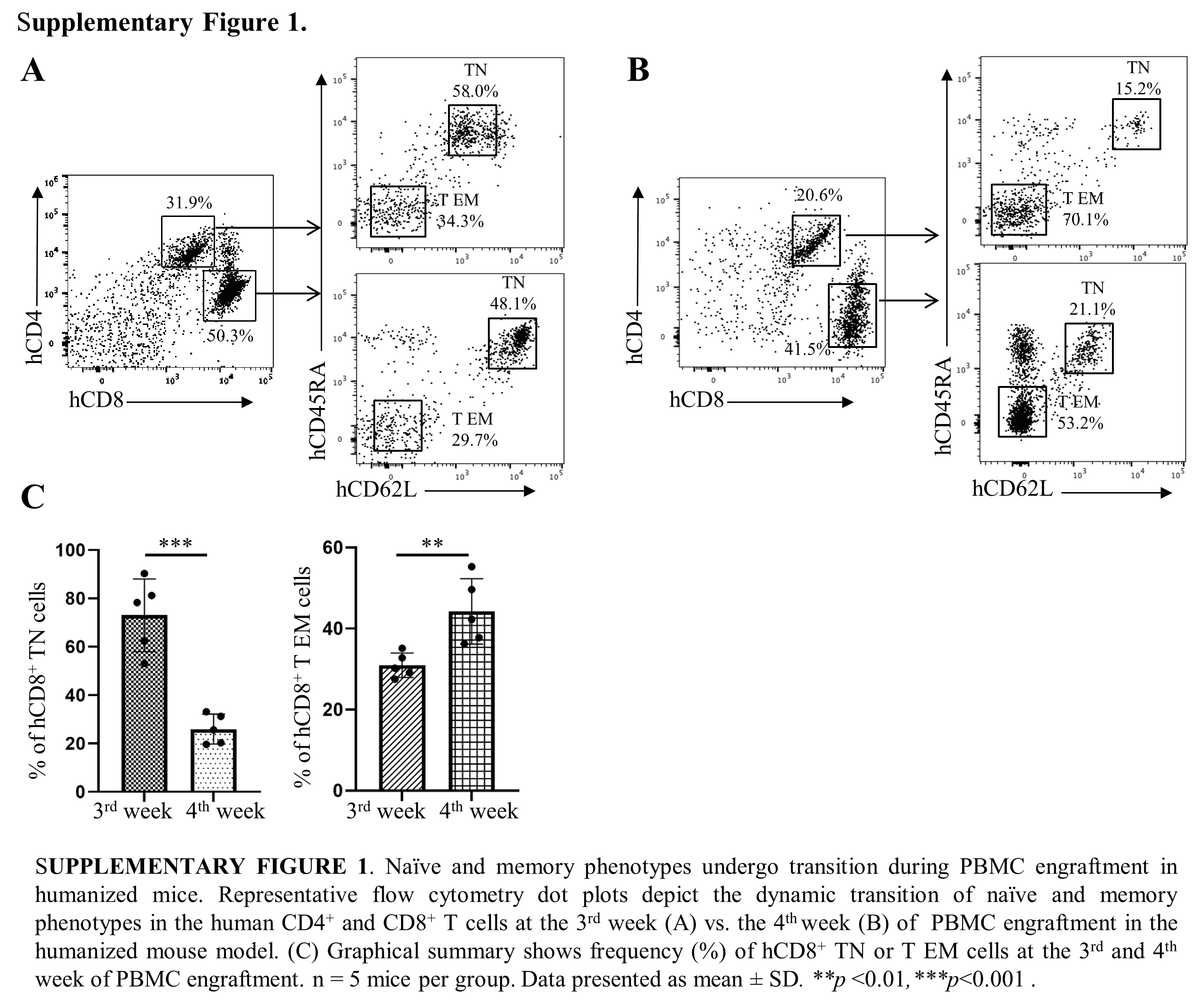

Supplement: Supplementary file 1 [file Image_1.tif]
